# Supplementary material for: Heart rate variability over the decades: a scoping review
Source: PeerJ. 2025 Apr 29;13:e19347. doi: 10.7717/peerj.19347 (PMC12047215; doi:10.7717/peerj.19347)
Supplement: Supplemental Information 1 [file peerj-13-19347-s001.pdf]

Supplementary Material

Supplementary table S1 contains information related to contemporary wearable devices with HRV measuring technology. The manufacturing year, if the device is tested against the gold standard and FDA approval information is taken from their respective sites. The supplementary table S2 contains a summary of all the articles that are included in the scoping review.

Table S1: Contemporary wearable devices/software with HRV measurement

Device	Year	Features	FDA Approval	Tested Against Standard
Polar S810	2001	HR, VO2max, HRV measurements	No	Yes
Polar S810i	2002	HR, predicts maximal oxygen uptake, HRV measurements	No	Yes
Actiheart (CamNtech Ltd., Cambridgeshire, UK)	2003	Combined HR and activity monitor	-	Mixed results, valid during rest
SUUNTO t6	2004	HR and HRV	No	Yes
Kubios HRV software	2004	Full featured HRV analysis, ECG, PPG, and HR data	No, categorized as low-risk	Yes, with good accuracy
Polar RS800	2005	Comprehensive athletic training features, HRV	No	Yes
irhythm Zio	2006	ECG Chest patch monitor	Yes	Yes
Polar RS800G3	2008	Comprehensive athletic training features, HRV	No	Yes
Actigraph wGT3X-BT (Actigraph, Pensacola, United States)	2009	HR monitor, sleep/wake monitor	-	Mixed results, valid during rest
Motorola Droid	2009	HRV measured using mobile camera with an app	No	Yes
Polar RS800CX	2009	Comprehensive athletic training features, HRV	No	Yes
Zephyr Bioharness 3	2011	Tracks HR, breathing rate, core body temperature, posture, and activity	Yes	Poor validity
iPhone 4S	2011	HRV measured using mobile camera with an app	No	Yes
Equivital EQ-02 (Equivital, Cambridge, UK)	2012	Comprehensive biometric monitoring	-	Mixed results, valid during rest
Polar H7	2012	Tracks HR and measures HRV	No	Mixed results
Ithlete PPG	2012	Measures HRV	No	Mixed results, valid during rest
Hexoskin	2013	ECG, HR, HRV, QRS events, HR Zones, HR Maximum, Resting HR, and HR Recovery	No	Yes
Ithlete Chest Strap	2013	HR, HRV	-	Limited research
HRV4Training PPG	2014	Mobile application for HRV insights	No	Yes
Polar V800	2014	HR and HRV	No	Yes
Empatica E4 (Empatica Inc, Boston, United States)	2015	PPG sensor for HRV, EDA sensor, skin temperature, motion-based activity	Yes	Mixed results, valid during rest
Apple Watch	2015	Tracks HR and measures HRV	Yes	Valid during all conditions

Continued on next page

Table S1 continued from previous page

Device	Year	Features	FDA Approval	Tested Against Standard
Whoop	2015	HR, HRV, blood oxygenation levels, breathing rate, and skin temperature	No	Yes
Oura Ring	2015	HRV, blood oxygen rate, body temperature, and sleep duration	No	Yes
AidlabTM (AidlabTM, Gdańsk, Poland)	2017	Personal insights, HRV analysis, activity tracking, skin temperature, heart monitoring, respiration rate	-	Mixed results, valid during rest
Polar H10 (Polar Electro UK Ltd., Warwick, UK)	2017	ECG HR monitor	No	Good to excellent validity
Biovotion Everion® (Biofourmis, Boston, United States)	2017	HR, respiratory rate, and blood oxygen saturation, skin temperature	Yes	Mixed results, valid during rest
Polar OH1	2017	Optical HR sensor, upper arm band	No	Yes, with good accuracy
Polar Vantage V (Polar Electro UK Ltd., Warwick, UK)	2018	HR and HRV	No	n/a
Fitbit Versa smartwatch	2018	Tracks steps, distance, sleep, stress, HR, and calories	Yes	Yes, with good accuracy
Garmin Vivosmart 4	2018	Tracks HR, sleep, stress, body energy, activity	No	Yes, with good accuracy
Garmin Fenix® 6X and 6S (Garmin Ltd., Southampton, UK)	2019	HRV tracking, VO2 max capacity, resting HR, Fast/slow HR notifications	-	Poor validity
Wahoo TICKR X	2020	Tracks HR, calories, workout in real time, chest strap	No	Yes
Apple Watch 6	2020	Tracks HR and notifies irregular rhythms, Measures ECG	Yes	Valid during all conditions
Fitbit Luxe	2021	Tracks HR, HRV, SpO2, breathing rate, skin temperature, sleep	Yes	Yes
Fitbit Charge 5	2021	Tracks HR, HRV, SpO2, breathing rate, skin temperature, sleep	Yes	Yes
Garmin Tactix® CharlieDelta (Garmin Ltd., Southampton, UK)	20182020	HR Monitor	Pulse Ox	Yes
Bittium Faros TM (Bittium, Oulu, Finland)	2018	ECG recordings, intelligent arrhythmia detection, HRV	Yes	Good to excellent validity
Bodyguard 2 (Firstbeat Technologies Ltd., Jyväskylä, Finland)	-	HR, HRV, Respiration rate, Blood oxygen saturation, and exercise	-	Yes
Elite HRV	2022	HRV software	No	Yes
Polar T31	-	HR and HRV	No	Yes, but lacks literary evidence

Table S2: All articles summary table

Reference	Year	Condition	HRV metrics	Confounding factors	Spectral analysis	Recording technique
[1]	1990	Acute myocardial infarction(HRV$\downarrow$)	An instantaneous change in HR can be expressed as the ratio (min RR)/(max RR)20 (e.g. the Valsalva ratio), or as the difference (max RR)-(min RR), SDNN	Age, gender, infarct site, ejection fraction, and beta-blocker treatment	Spectral Analysis and Autocorrelation	Holter records 24-hr, 24-hr ECGs
[2]	1993	Sudden death, Congestive heart failure, CHF, Diabetes, Alcoholic cardiomyopathy	SDNN, SDANN, RMSSD, SDNNIDX, PNN50, Baseline width, PSD, HF, LF, ULF, VLF, TP.	Medications	Fast fourier transform(FFT)	Holter recordings, 24-hr ambulatory ECG.
[3]	1995	Coronary artery disease (HRV$\downarrow$), Mitral valve disease(HRV$\downarrow$), Sleep apnea, Hypertensive patients (HRV$\downarrow$)	SDNN, SDANN, SD index, PNN50, RMSSD, PSD, LF, HF	Age (HRV$\downarrow$), Exercise, Respiratory cycle, Blood pressure, Circadian variations, Intervention programs, Behavioral, Physiological, occupational and environmental factors	FFT	Holter recordings
[4]	1995	Deep breathing, Mortality, Sudden cardiac arrest, Myocardial infraction, Congestive heart failure, Diabetes, Renal disease, Hypertention, Autonomic neuropathy, Sudden death syndrome	SDANN, 24-hr SD, SD, RMSSD, RR50, %RR50	Exercise	FFT and Autoregressive (AR)	Holter tapes
[5]	1996	Ischemic heart disease(LF/HF $\uparrow$), Myocardial infarction, Essential arterial hypertension (LF $\uparrow$), Chronic heart failure (PNS $\downarrow$)	SDRR, HF, LF, VLF	Posture (tilt and rest), Arterial pressure variability	FFT and AR modelling	Holter tapes
[6]	1997	Diabetic Neuropathy, Heart Transplantation, Congestive Heart Failure, Left Ventricular Hypertrophy, Risk Stratification after Myocardial Infarction, Ventricular Arrhythmias	SDNN,SDANN,TINN,PNN50,RMSSD, PSD, HF,LF	Not Available	Not Available	ECG recordings and Ambulatory ECG
[7]	1998	Acute Myocardial Infarction, cardiac transplantation, Congestive heart failure, Cardioneurogenic syncope, Diabetic autonomic neuropathy, Depressive disorder, Ejection fraction	SDNN, triangular index, SDANN, RMSSD, PSD, HF, LF, ULF, VLF, PNN50, TINN, SDSD, NN50 Count, Differential index, logrithmic index, LF/HF	Not Available	Not Available	Holter recordings
[8]	1999	Mortality, Arrhythmic death, Coronary events, Heart failure, Acute myocardial infarction	SDNN, Geometric triangular analysis, Poincare plot analysis, Fractal analysis methods	Not Available	Not Available	Holter recordings and 24-hr ambulatory recordings

Continued on next page

Table S2 continued from previous page

Reference	Year	Condition	HRV metrics	Confounding factors	Spectral analysis	Recording technique
[9]	1999	Myocardial infraction, Sudden death, Malignant ventricular arrhythmias, Morbidity and mortality, Congestive heart failure, Diabetes	SDNN, SDANN, SDNNIDX, PNN50, RMSSD, HF, LF, VLF, ULF, TP, LF/HF	Age	Not Available	24-hr Holter recordings
[10]	1999	Not Available	SDNN, Triangular index, SDANN, RMSSD, coefficient of variance of RR interval (CV-RR), LF, HF, LF/HF	RSA, MWSA (Mayer-wave-related sinus arrhythmia)	FFT and maximum entropy method (MEM)	ECG, Tachogram
[11]	2002	Mental and physical stress, Verbalization during mental activity noticeably influences, Congestive heart failure, Post-infarction arrhythmias, Hypertension, Diabetic patients, Acute myocardial infarction	SDNN, RMSSD, PNN50, SDANN, Histogram of the NN-intervals, Lorentz-plot (or Poincaré-plot)	Aging(lower in women compared to men)	FFT and AR Modeling	Holter monitors
[12]	2002	Mortality, Coronary artery diseases, Hypertention, Congestive heart failure, Sudden cardiac death	SDNN, Triangular index, HF, LF, LF/HF, Fractal correlation	Pharmacological interventions	Not Available	Holter systems
[13]	2003	Not Available	Not Available	Age, Gender, Genetic factors Smoking, Caffeine, Posture, Physical activity, Respiratory activity, Blood pressure variability, Central mechanisms (emotion, stress, sleep, mental activity, arousal), Arterial baroreflex SNS, PMS, Humoral factors (plasma catecholamines, thyroid hormones), Pharmacological treatment (sympatholytic agents, digitalis, antiarrhythmic drugs)	Not Available	Not Available
[14]	2005	All-Cause or Cardiac Mortality After myocardial infraction, Theraputics	SDNN, SDANN, ASDNN (index) , RMSSD, NN50, PNN50, Night-day difference, LF, HF, VLF, ULF, Power law slope, The short- and long-term fractal-scaling exponent, and SD12, Poincare plots	Not Available	FFT and AR techniques	ECG recordings

Continued on next page

Table S2 continued from previous page

Reference	Year	Condition	HRV metrics	Confounding factors	Spectral analysis	Recording technique
[15]	2006	Blood pressure, Myocardial infraction (SNS↑ and PNS↓), Nervous system, Diabetes (HRV all indices↓), Cardiac arrhythmia, Renal failure, Brain damage, Depression (↓all cyclic changes), Diabetic neuropathy (PNS↓, Power spectrum indices↓)	SDNN,SENN, SDSD,RMSSD, NN50,pNN50, RR triangular index, TINN, Poincare geometry, Correlation dimension analysis, Detrended fluctuation analysis, Largest Lyapunov exponent, Approximate entropy, Hurst exponant (H), Sample entropy (SampEn), Recurrence plots	Gender (HRV↓ with age and variation is more in the case of females than men.), Age, Smoking(↑ SNS, ↓PNS), Drugs, Alcohol (HRV ↓), Infants, Sleep	FFT, Short-time FFT, AR method, Higher order spectra, Wavelet transform, and Continuous time wavelet transform analysis	ECG
[16]	2006	Myocardial Infarction, Heart Failure, Sudden death	SDNN, SDANN, PNN50, RMSSD, PSD, LF, HF, LF/HF, detrended fluctuation analysis(DFA), approximate entropy	Cardiovascular Drugs	FFT	Holter monitor
[17]	2009	Physical exercise, Essential arterial hypertension (LF↑), Myocardial infarction, Transient myocardial ischemia, Congestive heart failure	LF/HF	Posture (tilt and rest)	FFT algorithm and AR modeling	Not Available
[18]	2011	Myocardial infarction, Diabetes, Hypertension, Heart failure, Fetal stress, Acute myocardial ischemia, Ventricular fibrillation	SDNN, SDANN, SDNNi, RMSSD, NN50, PNN50, Triangular index, TINN, TP, ULF, VLF, HF, LF, Lf nu, Hf nu, LF/HF, Chaos theory	RSA, Baroreflex, Exercise, Postural changes, Obesity, Work stress, Smoking	Not Available	ECG, kymograph, and Holter device
[19]	2012	Diabetes, Cardiovascular risk factors, Atherosclerosis, Coronary artery disease, Ischemic sudden death, Coronary instability, Heart failure	SDNN, SDANN, RMSSD, SDNN Index, SDSD, NN50, pNN50, TP, VLF, LF norm, LF, HF norm, HF, LF/HF, ULF	Circadian pattern, Aging, Gender, Ethnicity	FFT and AR	Not Available
[20]	2012	Not Available	HF, LF, VLF, ULF, DFA, SDNN, RMSSD, SDANN, PNN50	Not Available	FFT	Holter monitor
[21]	2013	Diabetes, Myocardial infarction, Nonischemic cardiomyopathy, Heart failure, Endocrinopathies (mostly in diabetes), Sudden death, All-cause mortality, CHF, Cardiac autonomic dysfunction (CAN), Stroke, Multiple sclerosis, Muscular dystrophies, Parkinson's disease, Epilepsy	SDNN, RMSSD, PNN50, Triangular index, LF, HF, VLF, ULF, LF/HF, TP, DFA, Power law relationship analysis, Approximate entropy, PD2i	Age, Gender, Drugs	FFT	ECG recordings
[22]	2013	Not Available	Mean NN, mean HR Mean of all NN intervals, SDNN, RMSSD, SDSD, NN50/PNN50 (PNN10), ULF, VLF, LF, HF, TP, SD1, SD2, ApEn, SampEn	Not Available	Not Available	PPG signal, Finapres™system, and Impedance plethysmography system
[23]	2013	Not Available	HF, LF, LF/HF, TP, VLF	RSA, Blood pressure, Physically Active and Sedentary, Tilt	FFT and AR Technique	Not Available

Continued on next page

Table S2 continued from previous page

Reference	Year	Condition	HRV metrics	Confounding factors	Spectral analysis	Recording technique
[24]	2015	Myocardial infarction, Congestive heart failure, Subjects without evidence of heart disease	Entropy and regularity, Nonlinear dynamical systems and chaotic behaviour, Long-range correlation and fractal scaling, Short-term complexity, Standard time and frequency-domain parameters	Not Available	Not Available	Not Available
[25]	2016	Not Available	SDNN, SDANN, and SDNNi, RMSSD, PNN50, HF, LF, ULF, VLF, LF/HF, Fractal mathematics and chaotic dynamics	RSA, Arterial pressurre fluctuations, Tilt, Supine, Mayer wave oscillations	AR models and FFT	ECG
[26]	2017	Athlete performance gains, chronic pain patients	SDNN, SDRR, SDANN, SDNN Index (SDNNI), PNN50, RMSSD, Triangular index, TINN, ULF, VLF, LF peak, LF power, LF power nu, LF power %, HF peak, HF power, HF power nu, HF power %, LF/HF, Poincaré plot(S, SD1, SD2, AND SD1/SD2), Approximate entropy, Sample entropy, DFA, Correlation dimention (CD, D2)	Respiration, Paced Breathing, Age, Sex, Heart Rate, Health, position	AR and FFT	ECG, PPG, and Portapres
[27]	2017	Not Available	SDNN, SDANN, RMSSD, NN50, PNN50, Triangular index, Poincare-plot, PSD, TP, VLF, LF, HF, LF/HF, ULF, Entropy, Fractal Analysis	Sex (↓in women), Age (all parameters except ULF↓), Weight, Food intake(Dietary restriction: HF↑, LF↓), Ethnicity, Circadian effects(SDNN↑ at night in one study. Most parameters decreased at night), Sleep (REM sleep: TP, VLF, LF↑, LF ↓. Non-REM sleep: TP, VLF, LF ↓, LF↑. In light sleep, SDNN, LF and LF/HF values are similar to wakefulness. Sleep deprivation: LF↑, conflicting results), Regular exercise(SDNN, TP, HF, fractal dimension↑), drugs	FFT and Wavelet analysis	Not Available

Continued on next page

Table S2 continued from previous page

Reference	Year	Condition	HRV metrics	Confounding factors	Spectral analysis	Recording technique
[28]	2019	Not Available	SDNN, PNN50, RMSSD, TP, VLF, LF, HF, SD1,SD2	Position(Supine, Tilt/recovery, Sitting, Standing, Active), Gender	Not Available	ECG, Portable devices (Elite HRV, Finapress PPG cuff, GOW Shirt, HRV4Training PPG, iPhone 4S, Ithlete PPG, Ithlete Chest Strap, Motorola Droid, Polar H7, Polar RS800, Polar RS800CX, Polar RS800G3, Polar S810, Polar S810i, Polar T31 Advantage, Polar T31 Armband, Polar T31 Counter, Polar V800, SUUNTOt6)
[29]	2019	Cardiac Autonomic Function, Multiple organ dysfunction syndrome, Sepsis, Chronic heart failure, Chronic kidney disease, Atherosclerosis, Myocardial infarction, Cardiac death, Idiopathic dilated cardiomyopathy, Blood pressure	LF, HF, ULF, LF/HF, SDNN	Circadian rhythm	FFT, AR, Trigonometric regressive spectral (TRS) analysis, Multiple TRS analysis(MTRS), Yule-Walker method and Burg algorithm, Wavelet analysis, SPWVD	ECG and holter recordings
[30]	2019	Intensive care medicine, Multiorgan dysfunction syndrome, Sepsis, Inflammation, Cardiovascular disorders, Arrhythmias, Cardiac arrest, Neurological disorders	SDNN, SDANN, RMSSD, SDNNi, SDSD, NN50, pNN50, Triangular index (TINN), Differential index Logarithmic index, Total power, VLF, LF, LF norm, HF, HF norm, LF/HF, α, S, SD1, SD2, SD1/SD2, ApEn, SampEn, DFAα1, DFAα2, D2	Age, Gender, Resting heart rate, Recent physical activity, Illness, Arrhythmias, Ectopic beats	FFT analysis	ECG recordings
[31]	2020	Myocardial ischemia, Heart diseases, Heart failure	LF, HF, PSD	Respiration, Standing, Exercise, Blood pressure, Body position, Location and body position, Body and environmental temperature	Not Available	ECG and PPG
[32]	2021	Cognitive performance, Invidualized training	SDNN, RMSSD, HF, LF, ULF, LF/HF	Not Available	FFT	Holter ECGs(multi and single lead), Bittium FarosTM 360, Actiheart, Firstbeat Bodyguard 2, Aidlab, PolarH10, Equivital EQ-02, Biovotion Everion, PPG (Empatica E4, Polar Vantage V, GarminWatches, Actigraph wGT3X-BT, Oura ring, AVA bracelet, and Sosche Rhythm 24TM)

Continued on next page

Table S2 continued from previous page

Reference	Year	Condition	HRV metrics	Confounding factors	Spectral analysis	Recording technique
[33]	2021	Heart failure, Neuro-cardio parameter, Medicine, Morbidity and mortality, Regular monitoring of patients with critical injury	Not Available	Age, Gender, Circadian rhythm, Physical exercise, HR, Respiration, Inflammation and infection, Viral infection, sepsis, Inflammation, Genetic factors, social stress, Noise increase, Depression, Chronic stress, Body mass index, Coffee consumption, Smoking, Alcohol consumption and cigarette smoking, Alternative medicine	Not Available	Not Available
[34]	2021	Post-MI patients, Cardiac death, Estimation of sleep stage by HRV, Sleep apnea	Hypnogram, body motion (BM) by actigraphy, R-R interval (RRI), Power spectral density (PSD), and relative power of HF to the integrated width (w) of the frequency band around the HF peak.	Not Available	Not Available	Not Available
[35]	2021	Stress, Drowsiness, Video Game	SDNN, RMSSD, PNN50, LF, HF, LF/HF, SD1, SD2, ApEN, GSR std, GSR mean, RESP rate, VLF	Stress, Drowsiness, Awake, Fatigue	Not Available	Cardiotachometer, ECG (2 lead, 12 lead), PPG, Holter ECG, Polar RS800, GE light ECG, Clickholter ECG, RM6240B ECG, MP150 Biopac, Gear S PPG, eMotion Faros, eXim Pro, RespiBAN, Empatica E4, Biopac PPG, EDA, and UIM
[36]	2022	Medical Decision support, Cardiology, Mental health, Sleep health, Lifestyle, Intensive Care Unit (ICU) settings, Remote monitoring, Comorbid conditions, Oncology, Brain health, Diabetes, Epilepsy, Respiratory disease, Infant health	Not Available	Blood pressure, Addiction, Drug abuse	Not Available	ECG recording setup and PPG
[37]	2022	Cardiology, Diabetes Detection and Management, Sleep medicine, Stress, Guide endurance training, Mental health applications	SDNN, SDANN, RMSSD, Power(ULF, VLF, LF, HF), Peak(ULF, VLF, LF, HF), LF:HF, S, ApEn, DFA $\alpha1$, $\alpha2$	Not Available	Not Available	ECG, PPG, Accelerometers and gyroscopes, Holter monitors, iRhytm Zio, Medtronic, Polar OH1, Wahoo TICKR X, Fitbit Versa smartwatch, Garmin Vivosmart 4, Everion, Apple watch 6, Fitbit Luxe, Polar H7, and Zephyr Bioharness 3

Continued on next page

Table S2 continued from previous page

Reference	Year	Condition	HRV metrics	Confounding factors	Spectral analysis	Recording technique
[38]	2022	Smartwatch, mHealth	Not Available	Not Available	Not Available	Mobile ECG, Samsung, Fitbit, and Garmin
[39]	2023	Diabetes Detection, Sleep Apnea Detection, myocardial infarction, Cardiac arrhythmia, Hypertension, Renal failure, Psychiatric disorders, ANS Activity of Patients undergoing Weaning from Mechanical Ventilation, and monitoring of fetal distress and neonatal critical care	SDNN, RMSSD, NN50, PNN50, TINN, PSD, TP, HF, LF, ULF, VLF, LF/HF, Poincare plot, DFA, Recurrence plot, Chaos theory, Entropy	Alcohol	FFT and AR	ECG

References

[1] M. Malik and A. J. Camm, "Heart rate variability," *Clinical cardiology*, vol. 13, no. 8, pp. 570–576, 1990.

[2] P. K. Stein, M. S. Bosner, R. E. Kleiger, and B. M. Conger, "Heart rate variability: a measure of cardiac autonomic tone," *American heart journal*, vol. 127, no. 5, pp. 1376–1381, 1994.

[3] E. Kristal-Boneh, M. Raifel, P. Froom, and J. Ribak, "Heart rate variability in health and disease," *Scandinavian journal of work, environment & health*, pp. 85–95, 1995.

[4] M. J. Cowan, "Measurement of heart rate variability," *Western journal of nursing research*, vol. 17, no. 1, pp. 32–48, 1995.

[5] F. Lombardi, A. Malliani, M. Pagani, and S. Cerutti, "Heart rate variability and its sympatho-vagal modulation," *Cardiovascular research*, vol. 32, no. 2, pp. 208–216, 1996.

[6] J. Kautzner and A. John Camm, "Clinical relevance of heart rate variability," *Clinical cardiology*, vol. 20, no. 2, pp. 162–168, 1997.

[7] A. Stys and T. Stys, "Current clinical applications of heart rate variability," *Clinical cardiology*, vol. 21, no. 10, pp. 719–724, 1998.

[8] H. V. Huikuri, T. Mäkikallio, K. J. Airaksinen, R. Mitrani, A. Castellanos, and R. J. Myerburg, "Measurement of heart rate variability: a clinical tool or a research toy?," *Journal of the American College of Cardiology*, vol. 34, no. 7, pp. 1878–1883, 1999.

[9] P. Stein, PhD and R. Kleiger, MD, "Insights from the study of heart rate variability," *Annual review of medicine*, vol. 50, no. 1, pp. 249–261, 1999.

[10] H. Kobayashi, K. Ishibashi, and H. Noguchi, "Heart rate variability; an index for monitoring and analyzing human autonomic activities," *Applied Human Science*, vol. 18, no. 2, pp. 53–59, 1999.

[11] L. Hejjel and I. Gál, "Heart rate variability analysis," *Acta Physiologica Hungarica*, vol. 88, no. 3-4, pp. 219–230, 2001.

[12] F. Lombardi, "Clinical implications of present physiological understanding of hrv components," *Cardiac electrophysiology review*, vol. 6, pp. 245–249, 2002.

[13] G. Parati and M. Di Rienzo, "Determinants of heart rate and heart rate variability," *Journal of hypertension*, vol. 21, no. 3, pp. 477–480, 2003.

[14] R. E. Kleiger, P. K. Stein, and J. T. Bigger Jr, "Heart rate variability: measurement and clinical utility," *Annals of Noninvasive Electrocardiology*, vol. 10, no. 1, pp. 88–101, 2005.

[15] U. Rajendra Acharya, K. Paul Joseph, N. Kannathal, C. M. Lim, and J. S. Suri, "Heart rate variability: a review," *Medical and biological engineering and computing*, vol. 44, pp. 1031–1051, 2006.

[16] K. C. Bilchick and R. D. Berger, "Heart rate variability," *Journal of cardiovascular electrophysiology*, vol. 17, no. 6, pp. 691–694, 2006.

[17] N. Montano, A. Porta, C. Cogliati, G. Costantino, E. Tobaldini, K. R. Casali, and F. Iellamo, "Heart rate variability explored in the frequency domain: a tool to investigate the link between heart and behavior," *Neuroscience & Biobehavioral Reviews*, vol. 33, no. 2, pp. 71–80, 2009.

[18] G. E. Billman, "Heart rate variability–a historical perspective," *Frontiers in physiology*, vol. 2, p. 86, 2011.

[19] B. Xhyheri, O. Manfrini, M. Mazzolini, C. Pizzi, and R. Bugiardini, "Heart rate variability today," *Progress in cardiovascular diseases*, vol. 55, no. 3, pp. 321–331, 2012.

[20] M. A. Peltola, "Role of editing of r–r intervals in the analysis of heart rate variability," *Frontiers in physiology*, vol. 3, p. 148, 2012.

[21] I. Cygankiewicz and W. Zareba, "Heart rate variability," *Handbook of clinical neurology*, vol. 117, pp. 379–393, 2013.

[22] A. Schäfer and J. Vagedes, "How accurate is pulse rate variability as an estimate of heart rate variability?: A review on studies comparing photoplethysmographic technology with an electrocardiogram," *International journal of cardiology*, vol. 166, no. 1, pp. 15–29, 2013.

[23] G. A. Reyes del Paso, W. Langewitz, L. J. Mulder, A. Van Roon, and S. Duschek, "The utility of low frequency heart rate variability as an index of sympathetic cardiac tone: a review with emphasis on a reanalysis of previous studies," *Psychophysiology*, vol. 50, no. 5, pp. 477–487, 2013.

[24] R. Sassi, S. Cerutti, F. Lombardi, M. Malik, H. V. Huikuri, C.-K. Peng, G. Schmidt, Y. Yamamoto, D. Reviewers:, B. Gorenek, *et al.*, "Advances in heart rate variability signal analysis: joint position statement by the e-cardiology esc working group and the european heart rhythm association co-endorsed by the asia pacific heart rhythm society," *Ep Europace*, vol. 17, no. 9, pp. 1341–1353, 2015.

[25] A. E. Draghici and J. A. Taylor, "The physiological basis and measurement of heart rate variability in humans," *Journal of physiological anthropology*, vol. 35, pp. 1–8, 2016.

[26] F. Shaffer and J. P. Ginsberg, "An overview of heart rate variability metrics and norms," *Frontiers in public health*, p. 258, 2017.

[27] G. Ernst, "Hidden signals—the history and methods of heart rate variability. front public health. 2017 oct; 5: 265," 2017.

[28] W. C. Dobbs, M. V. Fedewa, H. V. MacDonald, C. J. Holmes, Z. S. Cicone, D. J. Plews, and M. R. Esco, "The accuracy of acquiring heart rate variability from portable devices: a systematic review and meta-analysis," *Sports Medicine*, vol. 49, pp. 417–435, 2019.

[29] K. Li, H. Rüdiger, and T. Ziemssen, "Spectral analysis of heart rate variability: time window matters," *Frontiers in Neurology*, vol. 10, p. 545, 2019.

[30] B. W. Johnston, R. Barrett-Jolley, A. Krige, and I. D. Welters, "Heart rate variability: Measurement and emerging use in critical care medicine," *Journal of the Intensive Care Society*, vol. 21, no. 2, pp. 148–157, 2020.

[31] E. Yuda, M. Shibata, Y. Ogata, N. Ueda, T. Yambe, M. Yoshizawa, and J. Hayano, "Pulse rate variability: a new biomarker, not a surrogate for heart rate variability," *Journal of physiological anthropology*, vol. 39, no. 1, pp. 1–4, 2020.

[32] K. Hinde, G. White, and N. Armstrong, "Wearable devices suitable for monitoring twenty four hour heart rate variability in military populations," *Sensors*, vol. 21, no. 4, p. 1061, 2021.

[33] R. Tiwari, R. Kumar, S. Malik, T. Raj, and P. Kumar, "Analysis of heart rate variability and implication of different factors on heart rate variability," *Current cardiology reviews*, vol. 17, no. 5, pp. 74–83, 2021.

[34] J. Hayano and E. Yuda, "Assessment of autonomic function by long-term heart rate variability: Beyond the classical framework of lf and hf measurements," *Journal of physiological anthropology*, vol. 40, no. 1, p. 21, 2021.

[35] S. Ishaque, N. Khan, and S. Krishnan, "Trends in heart-rate variability signal analysis," *Frontiers in Digital Health*, vol. 3, p. 639444, 2021.

[36] O. Faust, W. Hong, H. W. Loh, S. Xu, R.-S. Tan, S. Chakraborty, P. D. Barua, F. Molinari, and U. R. Acharya, "Heart rate variability for medical decision support systems: A review," *Computers in Biology and Medicine*, vol. 145, p. 105407, 2022.

[37] N. Alugubelli, H. Abuissa, and A. Roka, "Wearable devices for remote monitoring of heart rate and heart rate variability—what we know and what is coming," *Sensors*, vol. 22, no. 22, p. 8903, 2022.

[38] S. Gupta, A. Mahmoud, and M. R. Massoomi, "A clinician's guide to smartwatch "interrogation"," *Current Cardiology Reports*, vol. 24, no. 8, pp. 995–1009, 2022.

[39] S. K. Nayak, B. Pradhan, B. Mohanty, J. Sivaraman, S. S. Ray, J. Wawrzyniak, M. Jarzebski, and K. Pal, "A review of methods and applications for a heart rate variability analysis," *Algorithms*, vol. 16, no. 9, p. 433, 2023.
